# Supplementary figures and images for: B lymphocytes that enter the germinal center late preferentially differentiate into memory cells that recognize subdominant epitopes
Source: bioRxiv. 2025 Nov 1:2025.10.30.685663. Preprint. [Version 1] doi: 10.1101/2025.10.30.685663 (PMC12636482; doi:10.1101/2025.10.30.685663)

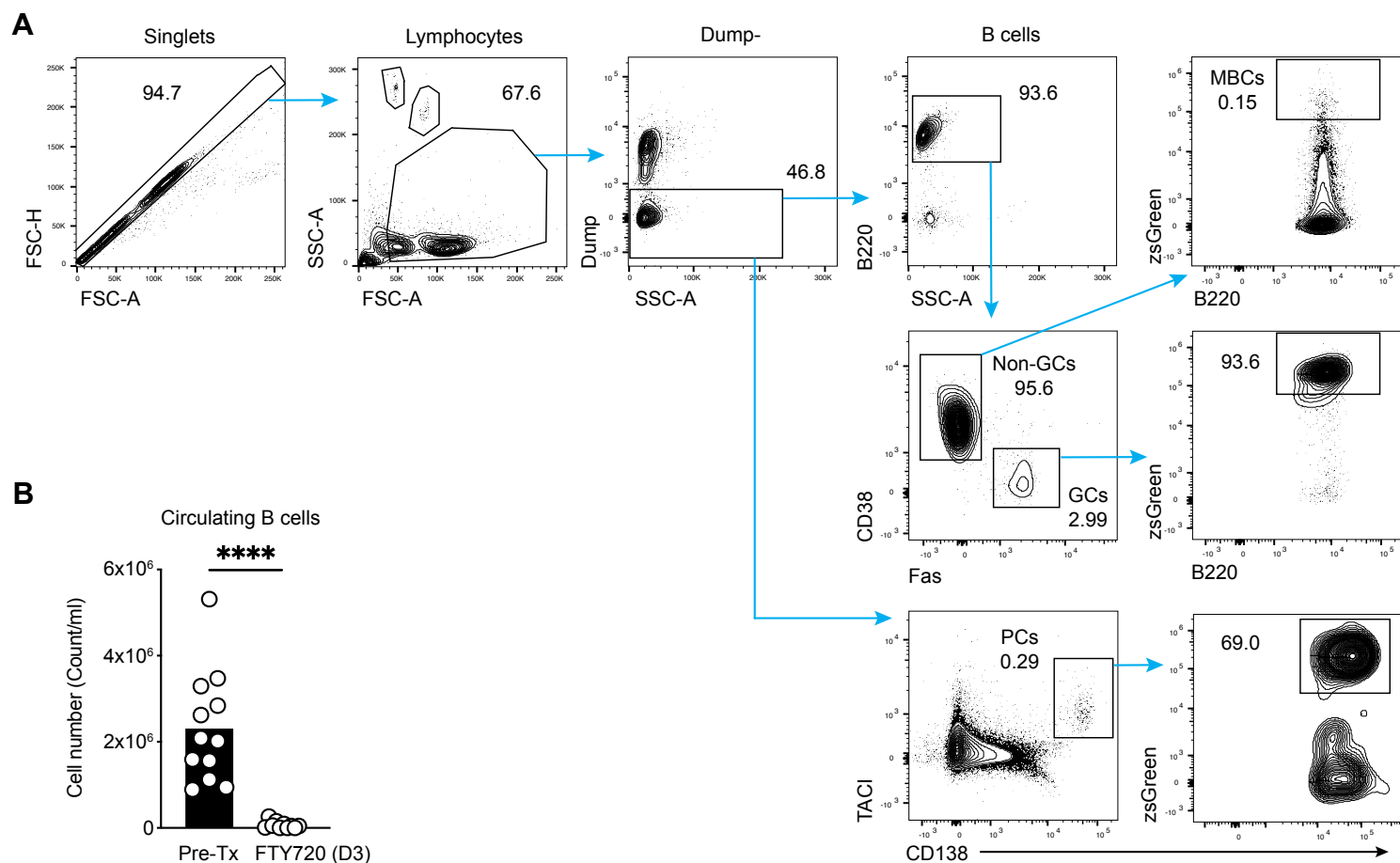

**Figure S1**

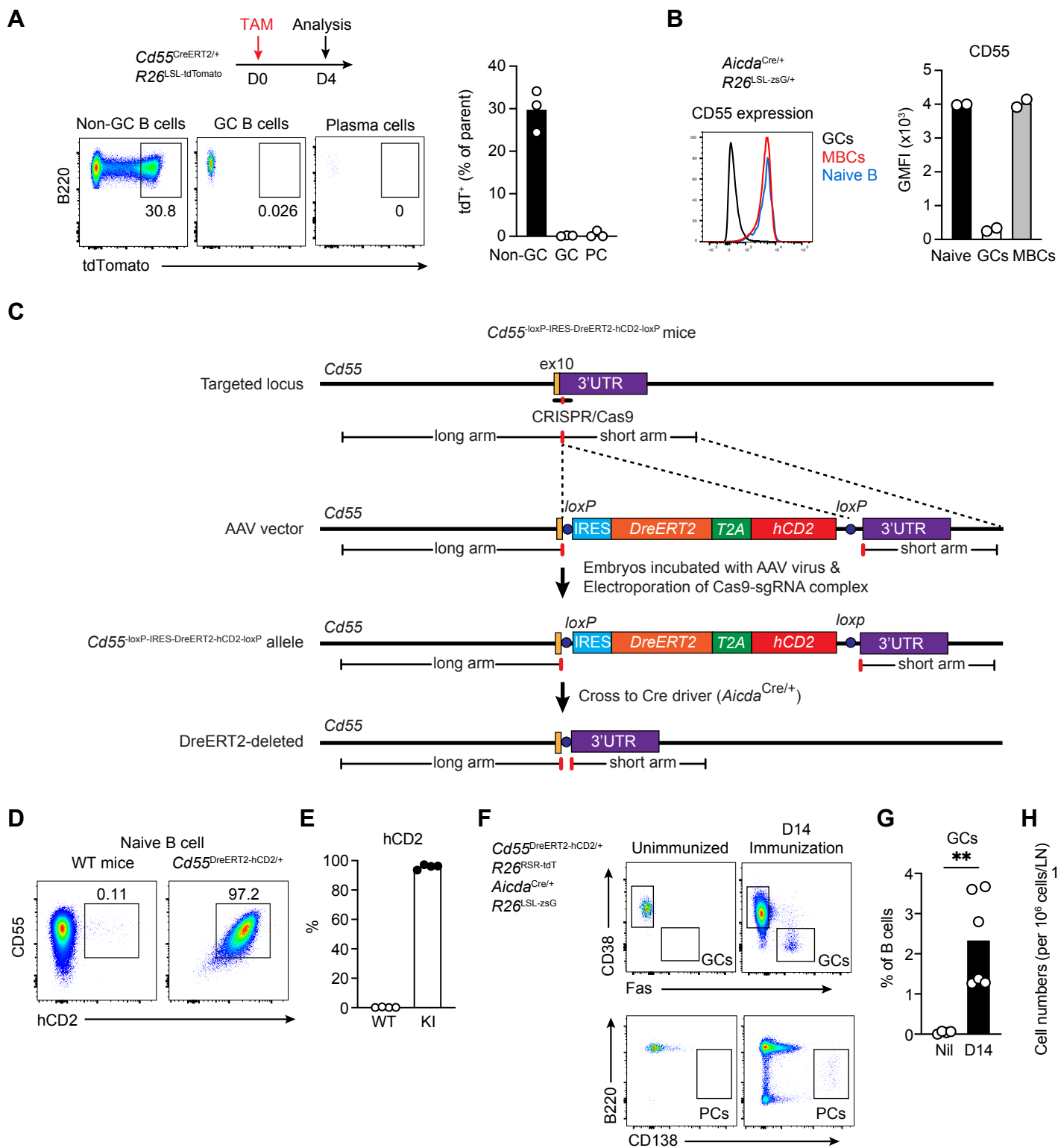

**Figure S2**

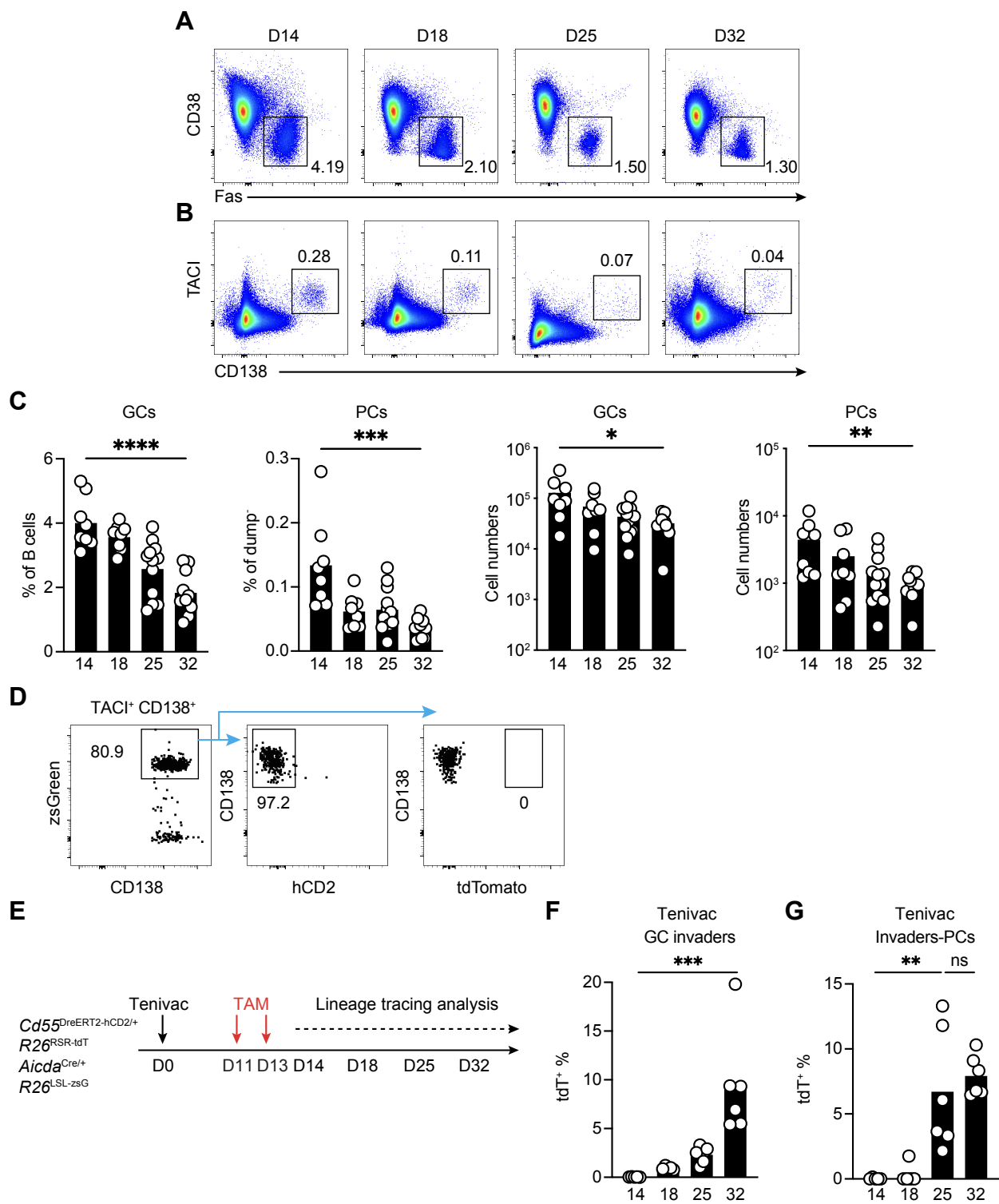

**Figure S3**

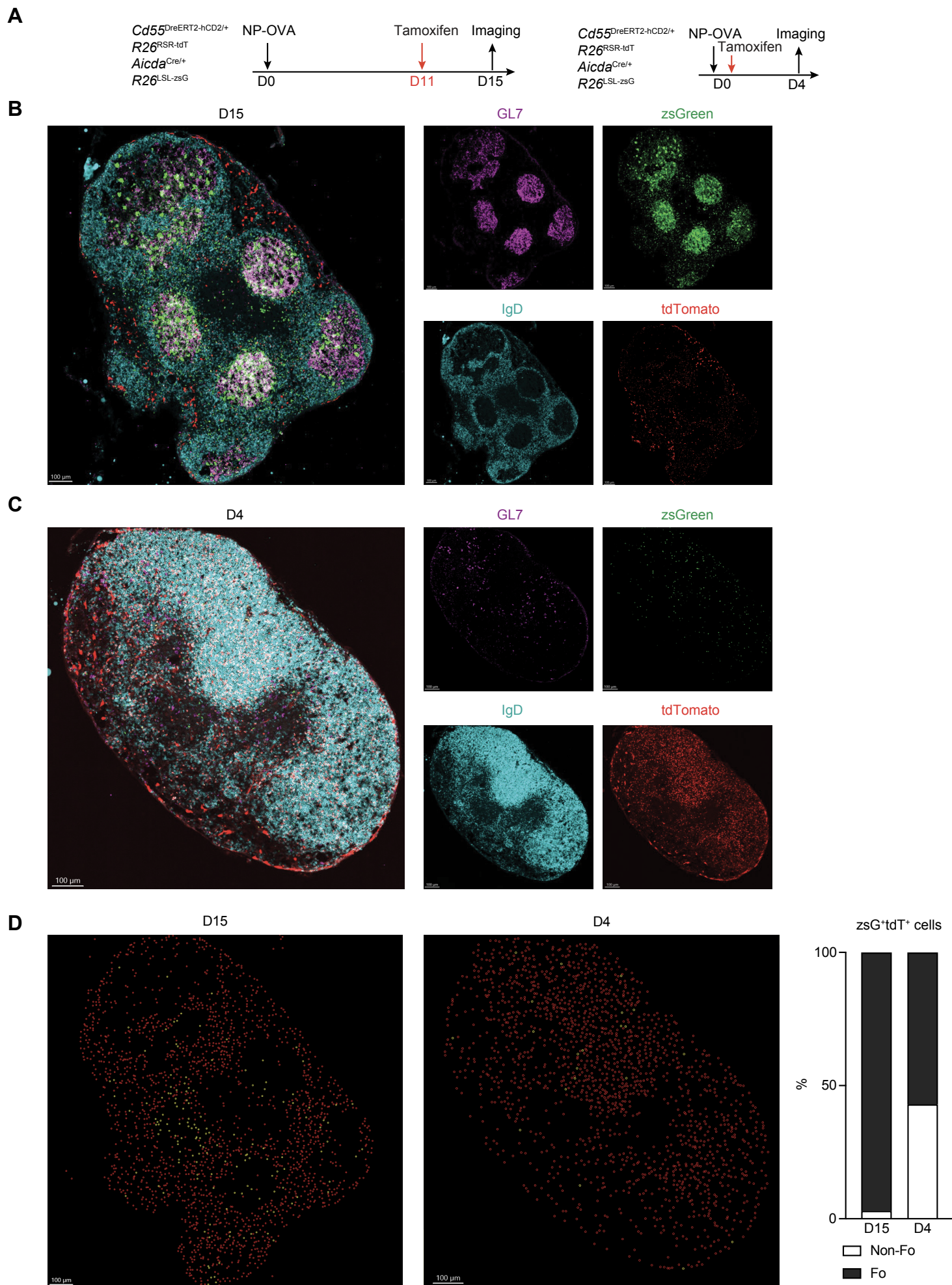

**Figure S4**

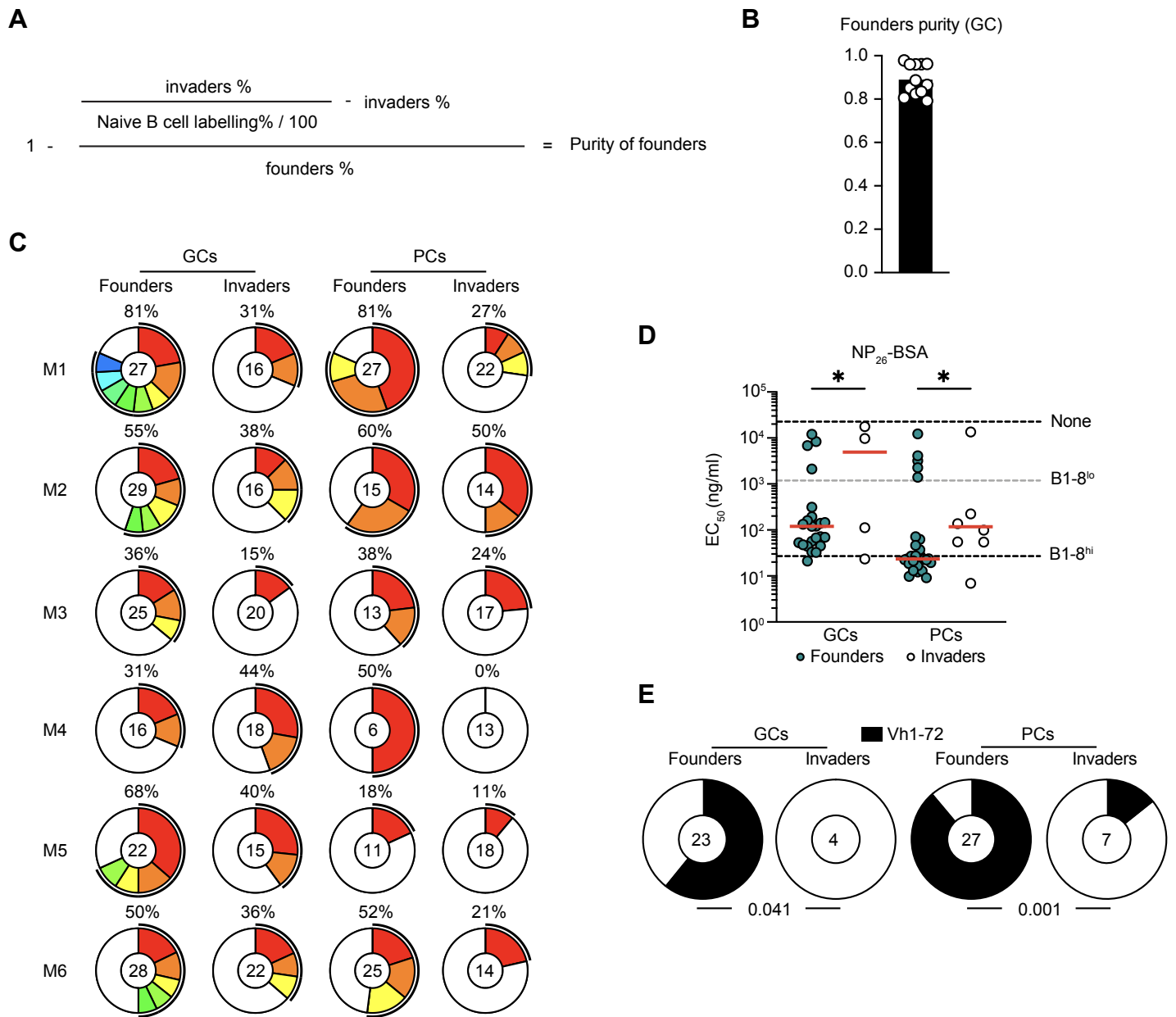

**Figure S5**

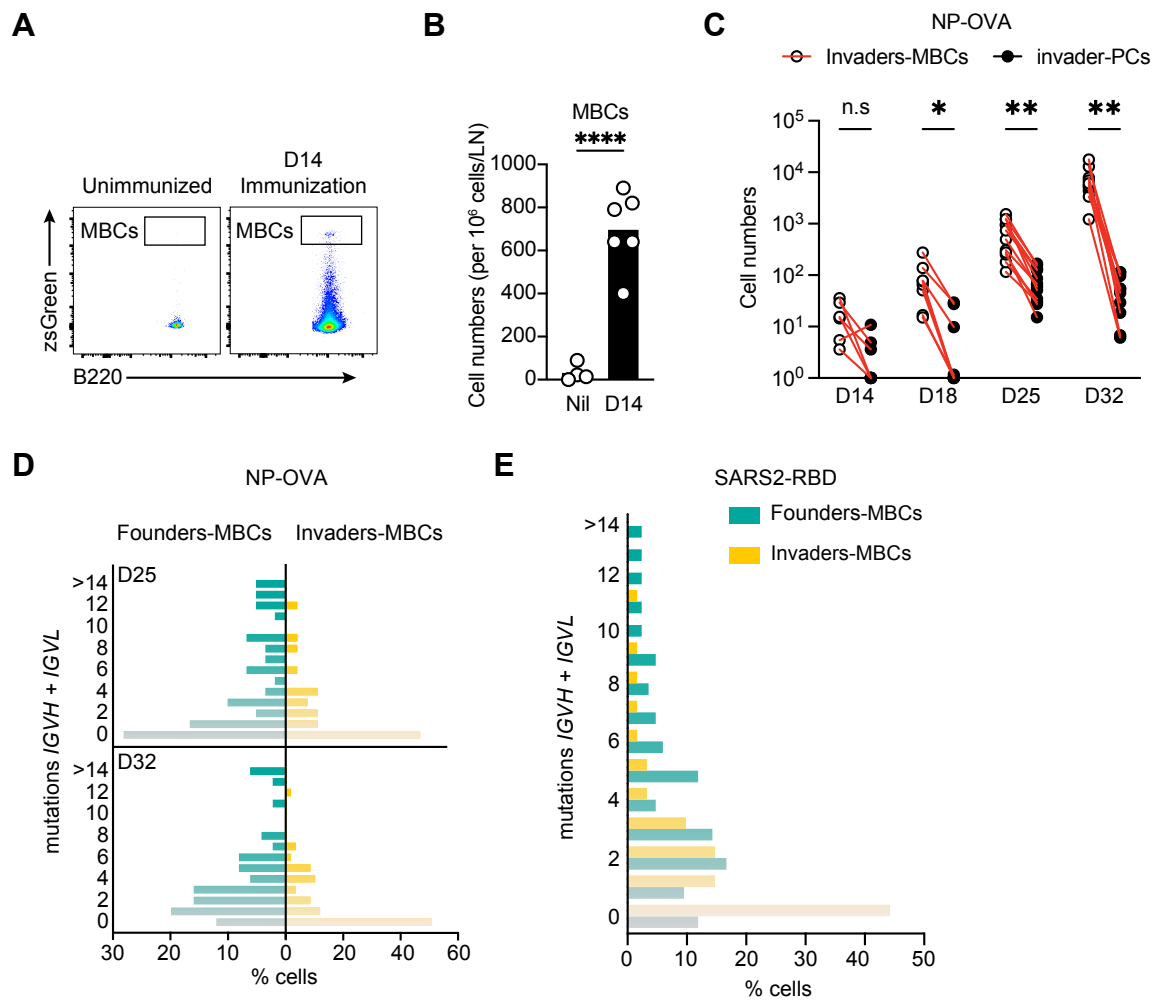

**Figure S6**

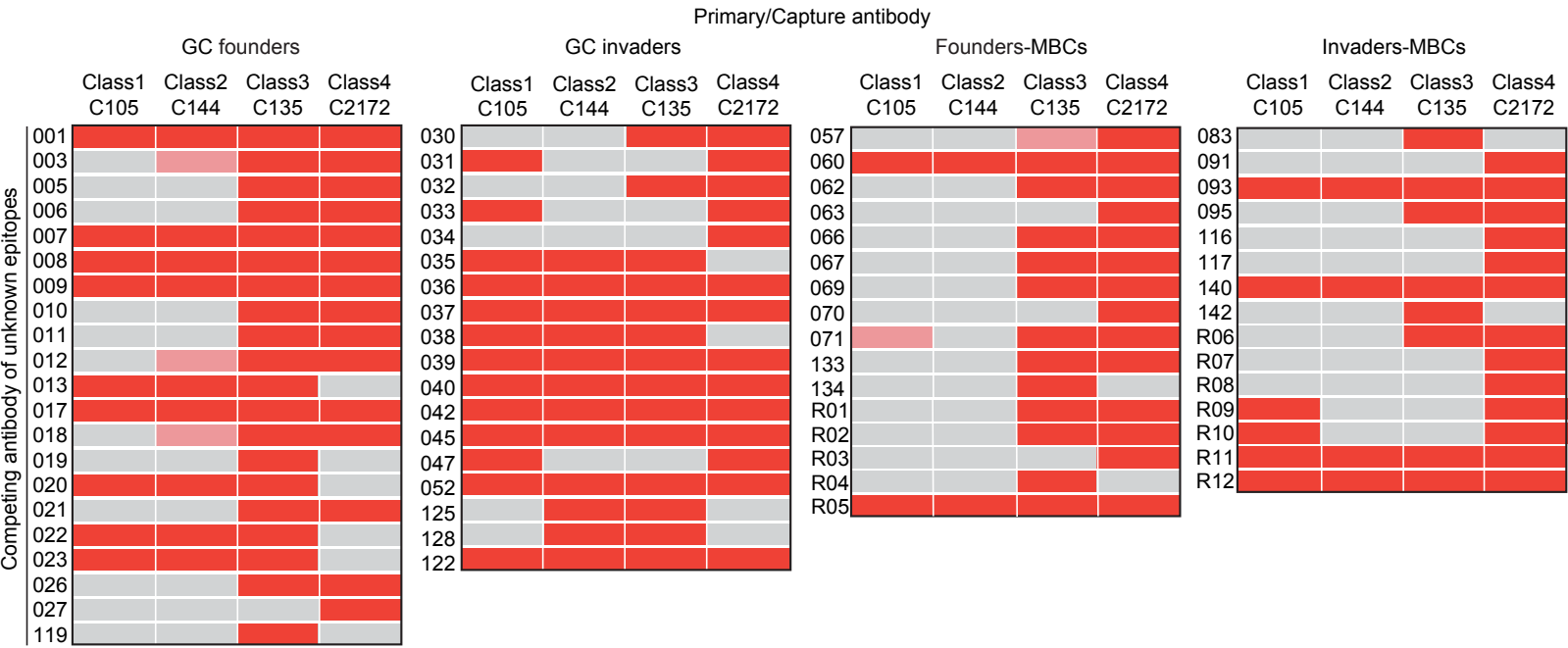

Figure S7

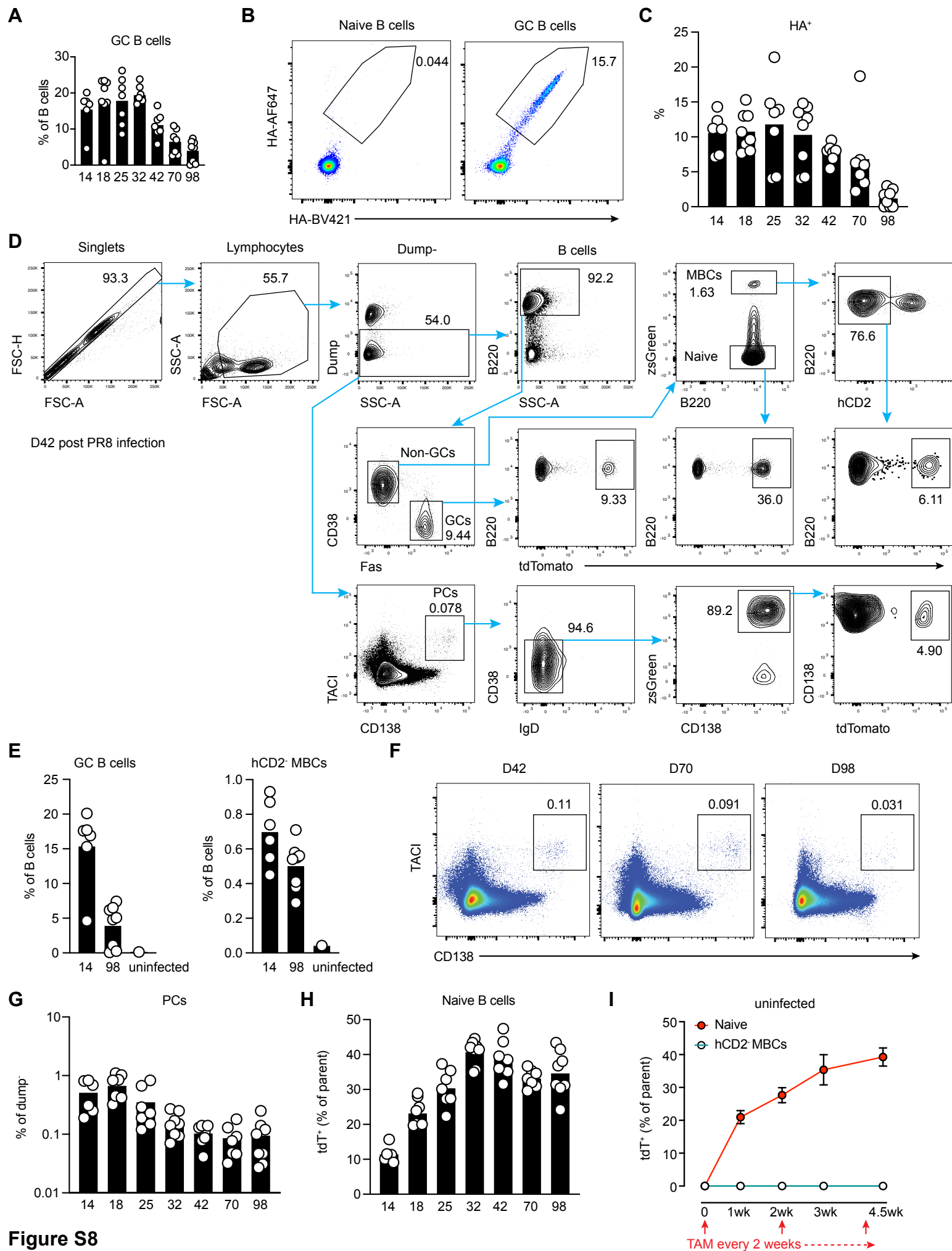

**Figure S8**

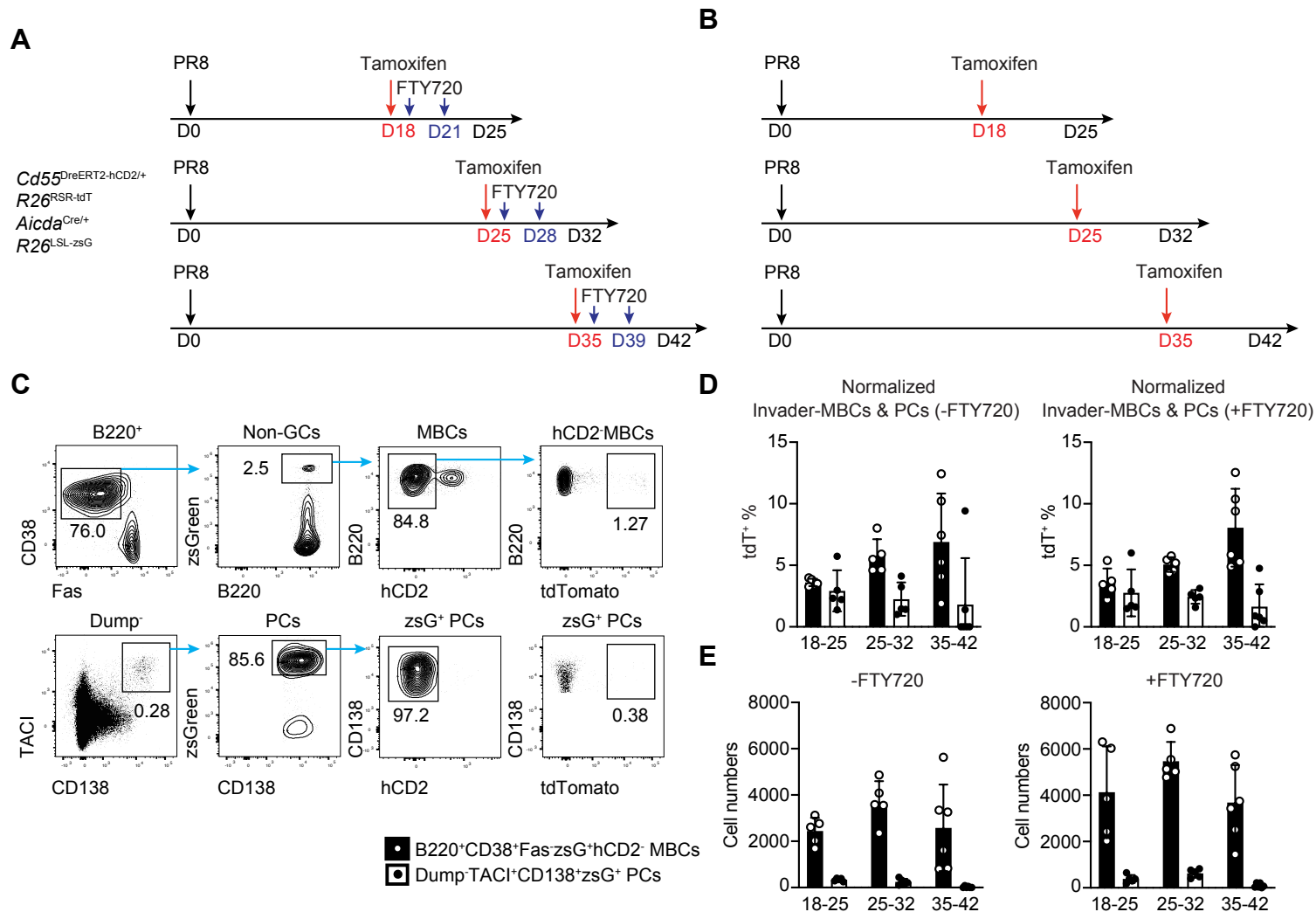

**Figure S9**

Supplement: Supplement 1 — Figure S1. Temporal dynamics of germinal center output, related Figure 1. (A) Gating strategy for zsGreen+ GC B cells, MBCs and PCs. Representative plots are from popliteal lymph nodes of S1pr2CreERT2/+ Rosa26LSL-zsGreen/+ mice immunized with NP-OVA and treated with tamoxifen. (B) Statistics showing the number of circulating B220+ B cells in the blood before and three days after FTY720 treatment in S1pr2CreERT2/+ Rosa26LSL-zsGreen/+ mice immunized with NP-OVA and treated with tamoxifen. Bar indicates mean. P values calculated using Mann-Whitney, ****P ≤ 0.0001. Figure S2. Generation of GC invader fate-mapping mice, related Figure 2. (A) Conditional labeling of follicular B cells in Cd55CreERT2/+ Rosa26LSL-tdTomato/+ mice. Mesenteric lymph nodes were analyzed 4 days after tamoxifen administration in naïve mice. Labeled tdT+ cells in B220+ CD38+ Fas− non-GC B cells, B220+ CD38− Fas+ GC B cells and B220lo CD138+ plasma cells were analyzed by flow cytometry. Left, representative flow cytometry plot. Right, statistics of tdTomato+ cells. (B) Examination of CD55 expression in AicdaCre/+ Rosa26LSL-zsGreen/+ mice. MBCs (B220+ CD38+ Fas− zsGreen+) and naïve B cells (B220+ CD38+ Fas− zsGreen−) in mesenteric lymph nodes were analyzed. Left, representative flow cytometry plot. Right, statistics of statistics of CD55 GMFI. (C) Targeting strategy and the configuration of the Cd55L-DreERT2-hCD2-L/+ allele. The mouse strain was produced in Nussenzweig Lab at The Rockefeller University and crossed to Rosa26RSR-tdTomato to create Cd55DreERT2-hCD2 Rosa26RSR-tdTomato conditional indicator mice. (D) Representative flow cytometry plot showing the hCD2 co-expression with CD55 in knock-in and wild-type control mice. (E) Statistics of hCD2 expression. (F) Representative flow cytometry plot showing GCs and PCs in popliteal lymph nodes obtained from CD55DreERAIDCre mice 14 days after or without immunization. (G) Graph showing the percentage of GC B cells in (F). (H) Graph showing the cell num [file media-1.pdf]
